# Unveiling the Druggable Landscape of Bacterial Peptidyl tRNA Hydrolase: Insights into Structure, Function, and Therapeutic Potential

**DOI:** 10.3390/biom14060668

**Published:** 2024-06-07

**Authors:** Surbhi Mundra, Ashish Kabra

**Affiliations:** 1 Department of Biochemistry and Biophysics, University of North Carolina at Chapel Hill, Chapel Hill, NC 27599, USA; surbhi_mundra@med.unc.edu; 2 Department of Molecular Physiology and Biological Physics, University of Virginia, Charlottesville, VA 22903, USA

**Keywords:** peptidyl-tRNA hydrolase, protein structure, Protein Data Bank, X-ray crystallography, NMR spectroscopy, esterase, antibacterial drug, tRNA, stalled ribosomes, puromycin, antimicrobial resistance (AMR)

## Abstract

Bacterial peptidyl tRNA hydrolase (Pth) or Pth1 emerges as a pivotal enzyme involved in the maintenance of cellular homeostasis by catalyzing the release of peptidyl moieties from peptidyl-tRNA molecules and the maintenance of a free pool of specific tRNAs. This enzyme is vital for bacterial cells and an emerging drug target for various bacterial infections. Understanding the enzymatic mechanisms and structural intricacies of bacterial Pth is pivotal in designing novel therapeutics to combat antibiotic resistance. This review provides a comprehensive analysis of the multifaceted roles of Pth in bacterial physiology, shedding light on its significance as a potential drug target. This article delves into the diverse functions of Pth, encompassing its involvement in ribosome rescue, the maintenance of a free tRNA pool in bacterial systems, the regulation of translation fidelity, and stress response pathways within bacterial systems. Moreover, it also explores the druggability of bacterial Pth, emphasizing its promise as a target for antibacterial agents and highlighting the challenges associated with developing specific inhibitors against this enzyme. Structural elucidation represents a cornerstone in unraveling the catalytic mechanisms and substrate recognition of Pth. This review encapsulates the current structural insights of Pth garnered through various biophysical techniques, such as X-ray crystallography and NMR spectroscopy, providing a detailed understanding of the enzyme’s architecture and conformational dynamics. Additionally, biophysical aspects, including its interaction with ligands, inhibitors, and substrates, are discussed, elucidating the molecular basis of bacterial Pth’s function and its potential use in drug design strategies. Through this review article, we aim to put together all the available information on bacterial Pth and emphasize its potential in advancing innovative therapeutic interventions and combating bacterial infections.

## 1. Introduction

**Figure 1:**
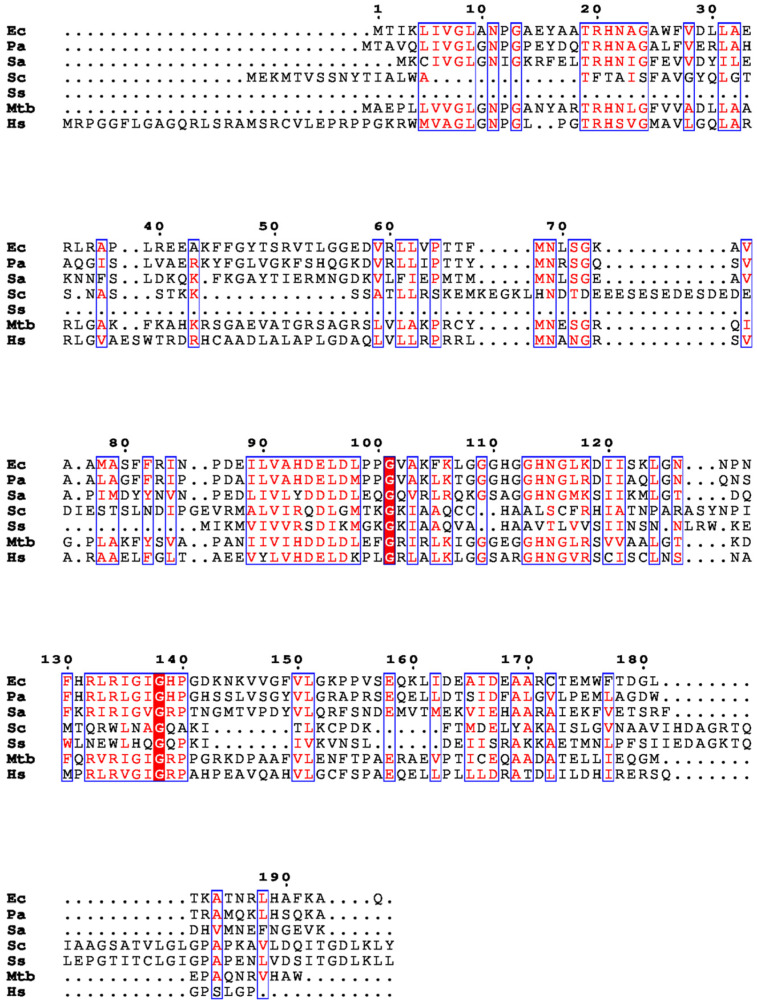
Multiple sequence alignment of Pth proteins from *E. coli*, *P. aeruginosa*, *S. aureus*, *S. cerevisiae*, *S. solfataricus*, *M. tuberculosis*, and *H. sapiens* is shown.

Protein synthesis, a fundamental and intricate process in all living cells, is governed by a series of well-coordinated molecular events. Protein synthesis or translation needs a complex machinery including messenger RNA (mRNA), transfer RNA (tRNA), ribosomes, and various protein factors. tRNA is a crucial adaptor molecule responsible for decoding genetic information carried by mRNA into the synthesis of proteins [1,2]. There are instances of peptidyl-tRNA drop off in the cells which could be attributed to various reasons, like minigene expression, frameshift mutations, truncated mRNAs, amino acid starvation, or tRNA starvation [3,4,5]. Recently, this process has been validated by mass spec analysis from the temperature-sensitive strains of *E. coli*. This process is deciphered to be an active quality control process in the cells [6]. These factors lead to the release of premature peptidyl tRNAs in the cell. The accumulation of these peptidyl tRNAs is lethal to the bacterial cells [7,8,9]. The fidelity and accuracy of protein synthesis depends on precise interactions among these various partners. Among the ensemble of various enzymes orchestrating these interactions, peptidyl-tRNA hydrolase (Pth) emerges as a key player in the final act of protein synthesis or the termination phase. This critical step is highly regulated, ensuring the release of the nascent polypeptide chain from the ribosome and the recycling of tRNA molecules for subsequent protein translation rounds [10,11,12]. The amino acid sequence alignment of Pth proteins, as shown in Figure 1, from different organisms indicates the protein to be highly conserved throughout the diversity of bacterial species and highlights the differences from the phylogenetically unrelated or distantly related species. Pth plays a pivotal role in the termination event by catalyzing the hydrolysis of the ester bond between the peptidyl moiety and tRNA [13]. Through this catalytic action, Pth liberates the completed polypeptide chain, allowing it to fold into its functional conformation and contribute to various cellular processes as well as maintain the free tRNA pool in the cellular environment.

**Figure 2:**
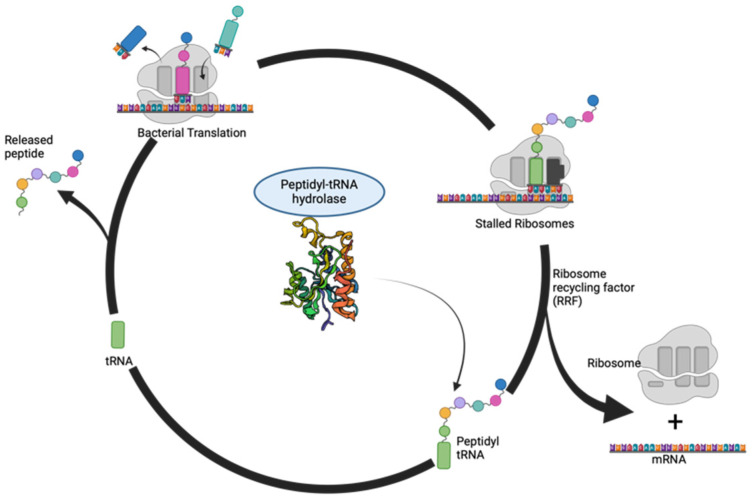
The release and cycling of peptidyl tRNA: The incomplete translation leads to the release of peptidyl tRNAs in the cell. The Pth protein enzymatically cleaves these peptidyl tRNAs, thus releasing free tRNAs in the cell for further rounds of protein synthesis. This figure was created with https://www.biorender.com/ (accessed on 1 June 2024).

A deeper exploration into Pth’s intricate structural and dynamic architecture is essential to understand its multifaceted role. The modular nature of Pth serves as a testament to its versatility as different regions within the enzyme contribute to substrate recognition, binding interactions, and catalytic activity. The catalytic core that is conserved across species represents the enzymatic prowess responsible for the hydrolysis of peptidyl-tRNA. The catalytic mechanism of Pth represents a finely tuned symphony of molecular events. A schematic for the hydrolysis of these peptidyl tRNAs in a bacterial cell is shown in Figure 2. As the ribosome approaches the termination codon, peptidyl-tRNA, carrying the nascent polypeptide chain, enters the enzyme’s active site. The highly dynamic nature of Pth becomes apparent as it undergoes conformational changes to accommodate the substrate. The hydrolysis of the ester bond is executed with exquisite precision, resulting in the separation of the peptidyl moiety from tRNA. The liberated tRNA is then poised for reuse, emphasizing the crucial role of Pth in the recycling of essential translation components [13].

Understanding the mechanistic intricacies of Pth not only sheds light on the termination phase of protein synthesis but also unravels the enzyme’s significance in maintaining translational fidelity. Mistranslation events, if left uncorrected, can lead to the production of aberrant proteins with potential deleterious consequences for cellular function. The vigilant action of Pth, facilitated by its highly dynamic nature, in preventing such errors underscores its indispensable role in the quality control mechanisms governing protein synthesis.

In the subsequent sections of this review, we will embark on a comprehensive exploration of peptidyl-tRNA hydrolase, delving into its structural features, substrate binding, and mechanisms of action. Furthermore, we will examine recent advances in our understanding of peptidyl-tRNA hydrolase and discuss its potential as a druggable target, opening avenues for therapeutic interventions in various pathological conditions. Overall, this review will eventually aid the researchers throughout the field to gain a concise understanding of the available Pth structures and knowledge about its interactions with its substrates. This review focuses on the use of Pth as a potential target and an alternative approach for combating the increasing incidences of antimicrobial resistance, or AMR.

## 2. Structural Insights from the PDB Database

**Figure 3:**
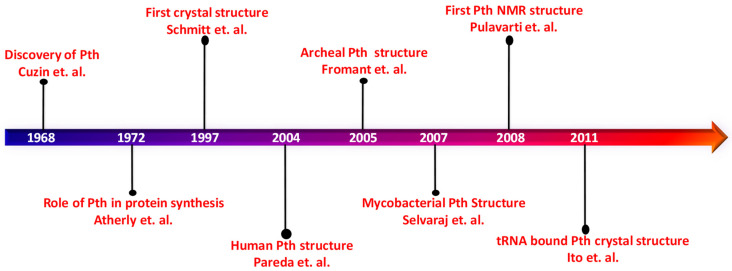
A timeline of the advancement in the knowledge of peptidyl tRNA hydrolase (Pth) [7,10,15,16,17,18,19,20].

The detailed examination of peptidyl-tRNA hydrolase’s structural features has been significantly enriched by the wealth of information available in the Protein Data Bank (PDB). Over time, numerous structures of Pth protein have been unraveled using an array of structural techniques such as X-ray crystallography and NMR spectroscopy, substantially enriching our comprehension of this protein. Despite the gene and its significance being recognized as early as the 1970s to 1990s, the earliest known structure of Pth, originating from *E. coli* (PDB ID: 2PTH), was documented in 1997 [9,13,14]. A timeline of the advancement of the knowledge on the structure and function of Pth protein is shown in Figure 3. The *E. coli* crystal structure, solved at 1.2 Å resolution, served as a seminal revelation, offering initial insights into the protein and its active site configuration [15].

**Figure 4:**
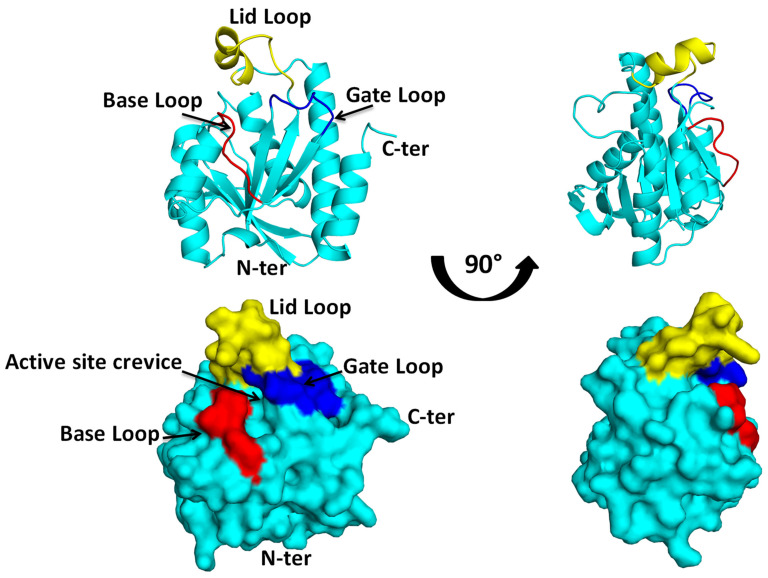
*E. coli* Pth structure (PDB ID: 2PTH) showing structural organization in bacterial Pth: **Top panel** is the cartoon representation of the protein which shows the arrangement of the helices and sheets in the tertiary structure of Pth (forming a closed cylindrical structure), with the base loop, lid loop, and the gate loop colored red, yellow, and blue, respectively. The **lower panel** shows the surface representation of the protein with all three loop regions, surrounding the active site crevice on the surface. The structures on the right side of the figure are the flipped view orientation of the protein by 90°.

Pth protein, resembling a compact globular entity, is characterized by a mixed beta sheet core enveloped by six alpha helices. While its secondary structure shares similarities with that of purine nucleoside phosphorylase (PDB ID: 1PBN), its functional disparity is noteworthy [21]. The organization of secondary structural elements conjures a closed cylindrical box-like formation, with helices and sheets harmoniously aligning to form a vessel-like structure. An intriguing feature is the formation of a complete vessel by all structural components, barring one helix, alpha 4, which assumes the role of a lid, as shown in Figure 4.

Scientific progress has stimulated an in-depth exploration and characterization of Pth proteins from various bacterial species after its initial discovery in *E. coli* [15]. Pth proteins originating from a spectrum of bacterial strains, including *Escherichia coli*, *Pseudomonas aeruginosa*, *Mycobacterium tuberculosis*, *Acinetobacter baumannii*, *Mycobacterium smegmatis*, *Vibrio cholerae*, *Salmonella typhimurium*, *Thermus thermophilus*, *Streptococcus pyogenus*, *Francisella tularensis*, *Burkholderia thailandensis*, *Staphylococcus aureus*, and *Klebsiella pneumoniae*, have been studied and characterized [15,16,17,22,23,24,25,26,27,28,29,30,31,32,33]. Analysis of Pth structures from these diverse microbial sources underscores a remarkable conservation of the protein’s global fold across different groups. Notably, all structures exhibit an identical overall protein architecture. The observed crevice on the protein’s surface has been delineated as the active site crevice, discerned from intricate structures of the protein with substrate analogs. This active site houses five crucial residues (three asparagines, one histidine, and one glutamate) that are highly conserved across species.

Progress in the understanding of the Pth protein’s structure has led to the delineation of specific regions within the protein, namely the base, gate, and lid loops, as shown in Figure 4. The base loop represents the foundation of the catalytic site pocket, while the gate loop is situated on the opposite side. Together, these loops form a gate that controls entry to the catalytic site pocket. The movement of these loops determines whether the gate is open or closed, ultimately regulating access to the substrate at the catalytic site. The active site crevice is covered by a highly flexible loop termed as the lid loop [33]. Each of these loops orchestrates distinct functionalities, facilitating substrate alignment and precise positioning at the binding site to optimize enzymatic activity. The secondary structural elements are packed, giving the protein a globular arrangement, and the active site crevice is surrounded by these three loops.

The elucidation of peptidyl-tRNA hydrolase structures extends beyond bacterial systems, encompassing eukaryotic organisms. The crystal structure of peptidyl-tRNA hydrolase from *Saccharomyces cerevisiae* (PDB ID: 2QA4) provides insights into the conservation of structural motifs across evolutionarily distant species [34]. Structural alignment of bacterial and eukaryotic peptidyl-tRNA hydrolases highlights the preservation of key residues involved in substrate binding and catalysis. This conservation underscores the fundamental importance of these structural features in maintaining the enzyme’s function across diverse cellular contexts.

**Figure 5:**
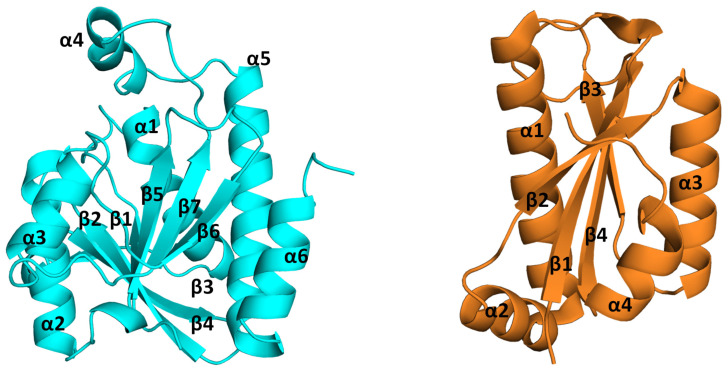
Cartoon representation of Pth proteins with labeled helices and sheets: (**Left**) Pth1 from *E. coli* (2PTH) comprising six alpha helices and seven beta strands, and (**Right**) Pth2 from *H. sapiens* (1Q7S) with four alpha helices and four beta strands.

The Pth proteins present in bacteria are referred to as Pth1, while those found in archaea, yeast, and eukaryotes are named Pth2. The vertical inheritance lineage of the *pth* gene is evident from the phylogeny study of the bacterial and eukaryotic Pth sequences [35]. Eukaryotes are known to contain orthologs of the Pth1 and Pth2 enzymes [36]. The gene deletion studies of both forms of Pth conducted on *S. cerevisiae* depict the non-essentiality of the protein, rendering the cell viable [35,37], while deletion of the Pth1 gene affected growth, suggesting the mitochondrial location of the protein [18,38]. These two Pth proteins, Pth1 and Pth2, differ in both sequence and structure [15,19]. On the one hand, the sequence alignment of Pth proteins from different bacterial species shows highly conserved regions along the whole sequence, while on the contrary, the Pth1 and Pth2 proteins do not show a significant sequence identity. The sequence comparison of Pth1 and Pth2 shows that Pth1 is longer (186–194 amino acids in length) than Pth2 (116–120 amino acids in length). Structurally, both proteins have an α/β fold, where the sheets form the central core of the protein surrounded by the helices. The Pth1 proteins possess seven mixed beta strands surrounded by six alpha helices, while the Pth2 proteins have four beta strands and four alpha helices. The Pth2 protein differs in its compactness compared to the Pth1 protein. A structural comparison of the arrangement of the secondary structural elements and difference in the compactness of the two proteins Pth1 and Pth2 can be seen in Figure 5. Pth2 proteins from *Homo sapiens*, *Methanocaldococcus jannaschii*, *Thermoplasma acidophilum*, *Sulfolobus solfataricus*, *Archaeglo busfulgidis*, and *Pyrococcus horikoshii* OT3 have been reported [18,19,39,40].

As we navigate the structural landscape of peptidyl-tRNA hydrolase through the lens of the PDB, it becomes evident that these structures serve as invaluable tools for deciphering the molecular intricacies of the enzyme. The elucidation of these structures not only refines our understanding of peptidyl-tRNA hydrolase’s catalytic mechanism but also opens avenues for structure-guided drug design, envisioning the development of compounds that could modulate the enzyme’s activity for therapeutic purposes.

## 3. Structural Insights from the Pth–Substrate Interactions

**Figure 6:**
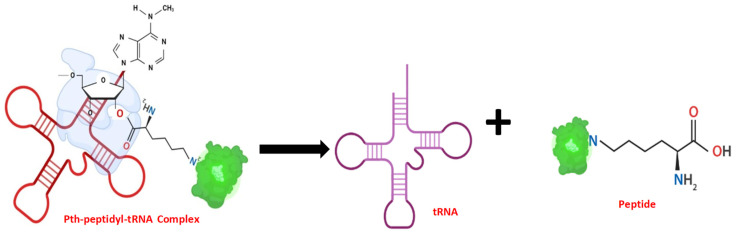
Representation of Pth’s action on its substrate N-blocked aminoacyl-tRNA: The ester bond between tRNA and peptide or N-blocked aminoacyl-tRNA (Green color represents the blockage of N-terminal, which can either be a fluorophore/molecule blocking the N-terminus of the peptide or a linked amino acid chain) is cleaved after the Pth action and free tRNA and N-blocked amino acid/peptide are released.

Peptidyl-tRNA hydrolases are essential enzymes and help in the rescue of stalled ribosomes. They are categorized as esterases and are responsible for the breaking of ester bonds between the peptidyl chain and tRNA. The protein rescues the cells from the conditions of tRNA starvation due to accumulation of peptidyl tRNA. The accumulation of peptidyl tRNA shortens the supply of free tRNA for further rounds of protein synthesis, thus affecting the process. Pth acts on these peptidyl tRNAs, cleaving the bond between the amino acid and tRNA and making free tRNA available to the translational machinery of the cell. N-blocked aminoacyl tRNA or peptidyl-tRNA with up to 3 to 4 amino acids forms the substrate for Pth [13]. A schematic of the mechanism of the binding of Pth to its substrate and its activity is shown in Figure 6.

**Figure 7:**
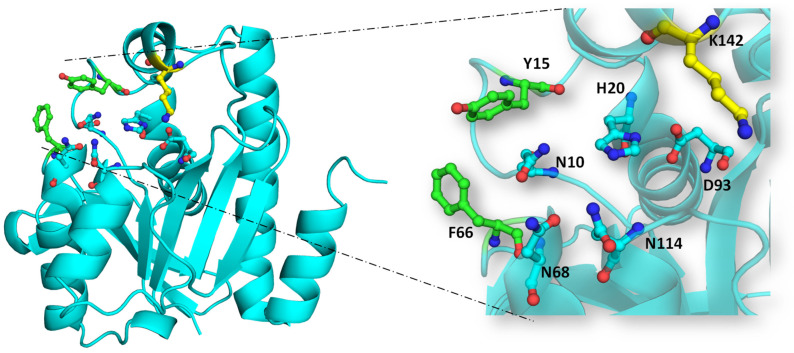
The orientation of active site residues in bacterial Pth: The active site residues N10, H20, D93, N68, and N114 form a crevice at the surface of the protein and shown in cyan color. Two aromatic residues Y15 and F66 are shown in green color that helps in stacking of the substrate whereas K142 of lid loop is shown in yellow color that interacts with CCA terminus of tRNA moiety. A zoomed-in view of the organization of the residues is shown on the right.

The substrate binding process in bacterial Pth is orchestrated by the coordinated action of various structural components of the enzyme, such as various loops, and its catalytic activity involves the complex chemical mechanism entailing hydrogen ion exchange. The X-ray structure of EcPth has one chain of the protein in its asymmetric unit. The structure shows the clamping of the C-terminal end of the symmetry-related protein chain to the active site crevice of the protein in its asymmetric unit. The C-terminal peptide _191_KAQ_193_ partially occupies the cleft at the surface of the protein, interacting with its main chain atoms. It might mimic the peptide moiety of the substrate for Pth. Specifically, the conserved asparagine at position N10 (in EcPth) engages with the main chain carbonyl of the penultimate residue of the peptidyl moiety. N68 and N114, meanwhile, bind to the carbonyl group of the C-terminal residue of the peptidyl chain attached to tRNA. Notably, N10 acts as a key residue in discriminating between aminoacyl-tRNA and N-blocked aminoacyl-tRNA or peptidyl-tRNA due to its interaction with the carbonyl of the penultimate residue, absent in unblocked aminoacyl-tRNA. The catalytic significance of H20 in EcPth is extensively documented, as this residue catalyzes the hydrolysis of peptidyl-tRNA, aided by D93. As shown in Figure 7, these five residues constitute a peptidyl binding site that interacts with the peptide moiety of the substrate, underscoring the intricate molecular mechanisms governing bacterial Pth’s function [15].

Recently, Ito et al. successfully crystallized the EcPth protein with the CCA-acceptor-TψC domain of tRNA. The crystals contained two molecules of the complex in one asymmetric unit. The structure of the complex revealed electrostatic interaction of the acceptor stem of the tRNA and the positively charged patch of the protein, and a shape complementary interaction between the minor groove of the TψC domain to the C-terminal helix of the protein. The lid loop region of the protein interacts with the 3′ CCA end of the tRNA molecule. The structure also affirms the placement of an adenine ring in the active site crevice of the protein and the involvement of two aromatic residues (F66 and Y15) in π–π interactions. The important information on the protein and tRNA interaction, depicted from this structure, is that all the main chain atoms of the substrate are involved in the interaction while the side chains point away from the protein, which implies a sequence-independent interaction between the two [20].

Various studies identified the important interactions between the Pth enzyme and peptidyl tRNA substrate. There are mainly three interactions:

- The positively charged patch of the protein and the acceptor site binding region of the substrate:

The presence of a region near the active site, which is rich in positively charged amino acids, makes it favorable for the stacking of the substrate at the crevice of the protein. This region comprises positively charged residues like K103, K105, and R133. The positive charge on these residues helps in the interaction of the protein with the 5′ phosphate of the substrate [40]. This positively charged patch on the surface of the protein interacts with the acceptor stem of the tRNA. These residues are conserved in different bacterial Pth proteins and have been shown to be important in substrate binding by site-directed mutagenesis [41,42].

B. The lid loop of the protein and CCA binding site of the tRNA:

The most flexible lid loop was expected to interact with the CCA site of the tRNA moiety. A conserved basic amino acid residue in the lid loop region (K142 in EcPth) was found to be in proximity to the backbone phosphates of C74 and the discriminator A73 nucleotides. Mutagenesis studies also showed that the k_m_ value of the K142A mutant was >5-fold larger in comparison to wild-type EcPth. So, it is assumed that there is an electrostatic interaction by which K142 interacts with the CCA terminus of the tRNA moiety and helps in stabilizing the peptidyl adenosine within the active site cavity of Pth [20].

C. C terminal of the protein and TψC domain of the tRNA:

The C-terminal region of the protein is highly variable and flexible among the bacterial species. This region is shorter in Gram-positive bacteria compared to that in Gram-negative bacteria. It includes the helix α6 that binds to the minor groove of the TψC domain of the tRNA. Two residues of the protein, N185 and H188, which are highly conserved among bacterial species, have been shown to interact with the substrate. Mutagenesis studies also confirmed the involvement of the H188 residue in substrate binding, as a 5.4-fold reduction in the k_cat_/k_m_ value was found in the H188A mutant of EcPth compared to that of the wild-type enzyme. The conserved asparagine, N185, interacts with G53 base and U(T)54 ribose via side-chain atoms. The N185A mutation in Pth decreased the k_cat_/k_m_ value by 5.7-fold compared to the wild type [20].

## 4. Structural Insights from Pth and Substrate Analog Interactions

Peptidyl tRNAs are the natural substrates for bacterial Pths. The mimics of this substrate, like puromycin, nucleotides, and acetylated and fluorescently labeled amino acylated tRNAs that bear the blocked NH of the amino acid, are used to study and decipher the mechanism of binding of the protein to its substrate. A lot of studies have been carried out with these substrate mimics to dig deeper into the orientation and mechanism of binding of the substrate to the enzyme. Some such studies are discussed in this section of the review. However, some of the earliest structural evidence of the Pth protein interacting with a peptide was revealed from the analysis of EcPth structures (PDB ID: 2PTH and 3VJR); some further studies using the substrate mimics were carried out to elucidate the mechanism of binding or interaction of the substrate to the protein.

In a study by Giorgi et al., the authors tried to elucidate the interaction mechanism between the substrate or substrate mimic of Pth with the protein using NMR spectroscopy combined with an in silico docking study. The group synthesized a compound, 3′-(L-[N, N-diacetyl-lysinyl)amino-3′-deoxyadenosine, mimicking the 3′ terminal adenosine of tRNA bound to an amino acid by an amide bond. The NMR titration studies of the protein with this compound showed binding in the residues of the active site cleft of the protein. The peak shifts of residues N10, F66, N114, M67, and N68 depict the binding of the molecule to the protein. The study also proposed the role of N114 in the catalysis of peptidyl tRNA [43].

In another study by the same group, they used a tRNA^His^ duplex and fragments to map its binding by NMR spectroscopy. From the study, it was concluded that the tRNA binds to the protein, as the peaks of the protein had decreased intensity with increasing concentration of the tRNA, indicating an increase in the resulting molecular weight of the sample. The chemical shift perturbations (CSPs) obtained for the active site crevice residues and the C-terminal helix of the protein were significant, indicating the hypothesized binding to be true [44].

Subsequent binding studies using Pth from *Pseudomonas aeruginosa* (PaPth) with a substrate mimic, an amino acylate-tRNA analog (AAtA), was performed by another group [29]. The AAtA comprises three moieties: O-tyrosyl, sugar, and adenine. Sugar and base (adenine or cytosine) moieties of the substrate mimic occupied the substrate binding cleft, while the O-tyrosyl moiety of the AAtA was positioned at the stacking position between Y17 and Y68. These three moieties of the AAtA occupied three distinct subsites in the PaPth molecule. The O-tyrosyl moiety is accommodated at subsite 1 (formed by Y17, Y68, M69, N70, and L152), while subsite 2 accommodates the sugar moiety (formed by N12, H22, N116, S148, V151, and L152). The remaining adenine moiety was placed at subsite 3, which is formed by L97, G113, G114, S144, and V147 of the PaPth molecule. This study shows a strong binding in the case of the AAtA due to the extra O-tyrosyl moiety in PaPth.

In a recent study on the interaction of puromycin, a peptidyl tRNA mimic, to the Pth proteins from *M*. *tuberculosis* and *E. coli*, it was found that the MtPth has a three-fold higher affinity to the substrate mimic compared to *E. coli*. MD simulation studies for both Pth proteins show a similar mechanism for their interaction, but MtPth demonstrates a preference for binding to the substrate through a water-mediated complex [45]. This was an interesting observation, and further exploration of this can open new avenues and insights for the field.

The availability of structures in complex with substrate analogs, such as the peptidyl-tRNA mimic, enhances our understanding of the enzyme–substrate interactions. Structures like that of a hydrolase from *Thermotoga maritima* in complex with its substrate analog (PDB ID: 5JIB) provide a snapshot of the enzyme engaging with its natural substrate, unraveling the spatial arrangement of residues critical for substrate recognition and binding [46].

## 5. Peptidyl-tRNA Hydrolase: Unraveling the Mechanism of Action

Peptidyl-tRNA hydrolase (Pth) stands as a sentinel at the culmination of protein synthesis, orchestrating the final act that liberates the nascent polypeptide chain from the tRNA molecule. Its catalytic prowess lies in the precise hydrolysis of the ester bond between the peptidyl moiety and tRNA, a reaction fundamental to the fidelity and efficiency of translation. Understanding the intricate mechanism by which Pth executes this hydrolysis unveils a molecular ballet that is essential for cellular homeostasis.

The journey of Pth begins as the ribosome approaches the termination codon, signaling the halt of polypeptide elongation. At this crucial juncture, the peptidyl tRNA, carrying the completed polypeptide, enters the catalytic cleft of Pth. The substrate of Pth comprises both an amino acid or peptide and a nucleic acid moiety or tRNA. This interaction is facilitated by specific regions of the Pth protein tailored for binding these distinct elements of the substrate, as discussed in earlier sections. This section describes the catalytic action of Pth.

The catalytic or active site cleft of Pth houses key residues that play pivotal roles in orchestrating the hydrolytic reaction. The hydrolytic reaction is the cleavage of ester bonds between peptide and tRNA and it is a step-by-step process that includes the coordination of various conserved residues, including catalytic histidine H20.

- Nucleophilic attack on ester bond: The conserved histidine residue (H20 in EcPth) within the active site forms a hydrogen bond with D93, enhancing the basicity of H20. This basic histidine then interacts with a proximal water molecule situated in the active site pocket and deprotonates it. This water molecule, stabilized at the catalytic site via interaction with N114, generates a hydroxyl anion that serves as a nucleophile. This anion attacks the carbonyl group of the ester bond between the peptide and tRNA. The interplay between N10 and N114 aids in optimal substrate positioning within the catalytic pocket.
- Stabilization of tetrahedral intermediate: The interaction between the hydroxyl anion and the ester bond forms a tetrahedral intermediate, further stabilized by interactions with two key asparagine residues, N68 and N114.
- Decomposition of tetrahedral oxyanion intermediate: The tetrahedral oxyanion intermediate decomposes through general acid catalysis, where D93, polarized by H20, facilitates the production of the peptide and free tRNA [20].

In addition to these active site residues, the orientation of Y15 and F66 influences the conformation of the region surrounding the crevice in the protein. Their positioning and interaction impact the stacking of the substrate at the active site crevice. These residues’ aromatic rings align closely at the opening of the substrate binding channel, where the substrate positions itself between them in the peptide-bound conformation. The dihedral angle of the F66 residue is influenced by the presence of any molecule or peptide moiety in the active site crevice. Notably, recent research underscores the significance of M71 within VcPth for maintaining the structural integrity of the Pth protein. Its interaction with critical active site residues, N14 and H24 of VcPth, optimally arranges these residues with respect to the substrate Pth, contributing to the protein’s compactness [47].

## 6. Druggability of Peptidyl-tRNA Hydrolase: Exploiting a Crucial Node in Translation Machinery

Drug discovery efforts are not new to the world and have been ongoing for a long time. To cure the diverse disorders and medical complications the world deals with, researchers have explored almost every biomolecule of the cell, including DNA, RNA, and proteins. Proteins are the most widely studied and targeted biomolecules for therapeutic development. The most explored proteins as drug targets include enzymes, kinases, and receptor proteins. Many antibiotics targeting different cellular processes like cell wall synthesis, DNA replication, and protein synthesis have been developed over time. Bacterial species can acquire antibiotic resistance by various means, like bacterial plasmids, chromosomes, or transposons [48]. The mode of antibiotic resistance in some cases can depend on the mechanism of action of the antibiotic. It can be either inactivation of the antibiotic or the alternation of its binding site. In some cases, the resistant gene can even change the cell permeability and in turn affect the internalization of drugs and the formation of biofilm [49,50,51]. Due to increase in the incidence of antimicrobial resistance, the antibiotic spectrum available for use is already converging, threatening the eradication of antibiotics [52].

Ever since the development of antibiotics, the currently prescribed antibiotics share a common scaffold targeting common bacterial life processes like DNA replication, cell envelope synthesis inhibition, protein synthesis inhibition, etc. [53,54,55,56,57,58]. These scaffolds lead to bactericidal effects. There has also been a lag in the development of new antibiotics, and filling this gap is the need of the current time. The requirement for new scaffolds for antibiotic design is alarming as cases of antibiotic resistance are increasing [59]. There has been a demand for new strategies to tackle AMR, which include auxiliary pathways rather than common targets, like quorum sensing, antibiotic adjuvants, the use of riboswitches, bacteriophages, and new protein targets.

Peptidyl-tRNA hydrolase (Pth) stands as a cornerstone in the final stages of protein synthesis, making it an attractive focal point for pharmacological intervention. Unraveling Pth’s susceptibility to drug targeting involves delving into its structural vulnerabilities, regulatory mechanisms, and the potential impact of small molecules or inhibitors on its catalytic function. The consequences of mistranslation events, if left unaddressed, can yield faulty proteins, posing significant threats to cellular function. Pth’s vigilant role in error prevention underscores its pivotal function in maintaining translation accuracy. For instance, investigations into temperature-sensitive Pth strains, bearing the G101D mutation, reveal the accumulation of peptidyl tRNAs at higher temperatures, leading to translational stalling and cellular starvation. The introduction of the G101D mutation deactivates bacterial Pth’s enzymatic activity, resulting in cell fatality. In summary, Pth emerges as a molecular conductor, orchestrating the final stages of protein synthesis with precision and evolutionary conservation. Its potential as a therapeutic target opens avenues for drug development and intervention strategies.

Detailed structural examinations of Pth, through crystallography and NMR studies, offer insights into regions amenable to pharmacological intervention. The catalytic domain, housing a critical histidine residue essential for peptidyl-tRNA hydrolysis, represents a prime target. Small molecules designed to interact with this active site could disrupt Pth’s enzymatic function. Additionally, regions involved in substrate recognition and binding provide alternative targets for interfering with the interaction of Pth with its substrate, potentially influencing enzyme functionality.

**Figure 8:**
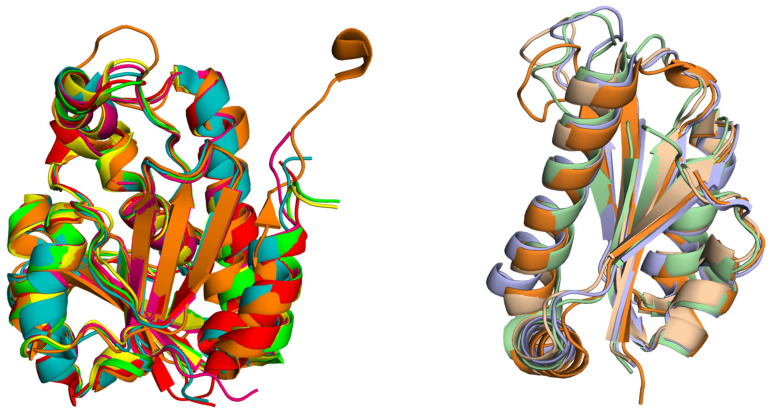
Overlay of Pth proteins: (**Left**) Pth1 proteins from *E. coli* (green), *A. baumannii* (cyan), *S. aureus* (orange), *K. pneumoniae* (yellow), *P. aeruginosa* (red) and *V. cholerae* (pink). The overlay shows the structural similarity among Pth1 from these different organisms. (**Right**) Pth2 from *S. sulfotaricus* (blue), *H. sapiens* (orange), *A. fulgidis* (green) and *T. acidophilum* (beige).

While bacterial Pth proteins share limited identity with their human counterparts (~33.5%), they present promising targets for antibiotic development. Inhibitors targeting crucial residues involved in substrate recognition may exhibit broad-spectrum activity against diverse organisms. However, achieving selective inhibition to minimize off-target effects is essential. Pth1, discovered in bacteria, exhibits a remarkably uniform structure across a wide range of organisms, whereas Pth2, identified in archaea and eukaryotes, shows a different overall fold that is also conserved across different species as illustrated in Figure 8.

Furthermore, post-translational modifications and protein–protein interactions modulating Pth’s function offer additional avenues for pharmacological intervention, impacting various cellular processes beyond protein synthesis. Careful consideration of specificity, toxicity, and off-target effects is vital in the development of Pth-targeting drugs. The pursuit of Pth as a therapeutic target holds promise for combating antimicrobial resistance (AMR), synergizing with existing antibiotics while minimizing eukaryotic cell toxicity. Its conservation across organisms and unique mode of action underscore its significance in tackling AMR.

In conclusion, Pth’s role in protein synthesis termination, its conservation across organisms, and its distinct mode of action make it an attractive target for therapeutic interventions, particularly in addressing antimicrobial resistance. The ongoing exploration of Pth’s druggability promises innovative strategies for combating AMR and advancing drug development efforts.

## 7. Discussion: Antimicrobial Resistance, Finding New Drug Targets and Inhibitor Design, and Charting the Future Course of Peptidyl-tRNA Hydrolase Research

Developing specific inhibitors in the context of antibiotics and antimicrobial resistance is crucial in addressing one of the most pressing global health challenges [60,61,62,63]. Antibiotics have revolutionized modern medicine, saving countless lives by combating bacterial infections. However, the widespread misuse and overuse of antibiotics have accelerated the emergence of antimicrobial resistance (AMR), rendering many once-effective drugs ineffective against bacterial pathogens [64,65,66,67]. Addressing AMR requires the development of novel antibiotics with improved efficacy, selectivity, and resistance profiles. Here is how the challenges associated with developing specific inhibitors relate to antibiotics and AMR:

- Selectivity: In the context of antibiotics, selectivity is paramount to avoid disrupting the beneficial microbiota while targeting pathogenic bacteria. Many traditional antibiotics have broad-spectrum activity, killing both harmful and beneficial bacteria, leading to dysbiosis and secondary infections [68,69,70,71]. Developing antibiotics with high selectivity for specific bacterial pathogens can minimize collateral damage to the microbiome and reduce the selective pressure for AMR [72,73].
- Resistance: Antibiotic resistance is a natural evolutionary response of bacteria to the selective pressure exerted by antibiotics [72]. Resistance mechanisms can arise through genetic mutations, horizontal gene transfer, or the acquisition of resistance genes from other bacteria [74,75]. Developing specific inhibitors that target essential bacterial functions or virulence factors can help minimize the emergence of resistance. Additionally, combination therapy strategies that target multiple pathways or utilize adjuvants to potentiate antibiotic activity can delay the development of resistance [76,77,78].
- Delivery Methods: Effective delivery of antibiotics to the site of infection is essential for achieving therapeutic concentrations while minimizing systemic exposure and toxicity. However, bacterial pathogens have evolved various mechanisms to evade antibiotic action, such as biofilm formation or efflux pump systems [79,80,81,82,83]. Developing innovative delivery methods, such as nanoparticles or localized drug delivery systems, can enhance the efficacy of antibiotics and overcome bacterial resistance mechanisms [84,85,86,87,88]. Targeted drug delivery strategies that exploit bacteria-specific surface markers or vulnerabilities can also improve the precision and efficiency of antibiotic treatment.
- D. Toxicity and Side Effects: Antibiotics can cause adverse effects ranging from mild gastrointestinal disturbances to life-threatening allergic reactions or organ toxicity. Developing antibiotics with improved safety profiles and reduced off-target effects is crucial for minimizing patient morbidity and mortality. Structure–activity relationship studies, pharmacokinetic optimization, and preclinical toxicity testing are essential steps in the development of safer antibiotics. Furthermore, strategies such as selective antimicrobial peptides or narrow-spectrum antibiotics can minimize the disruption of commensal microbial communities and reduce the risk of secondary infections [89,90].

By developing specific inhibitors that target bacterial pathogens with high selectivity, potency, and safety, we can mitigate the spread of AMR and ensure effective treatment options for infectious diseases in the future. Bacterial Pth is one of the novel targets for antibacterial development. The function of Pth is essential for maintaining the balance of proteins in the cell and ensuring that they are made correctly.

Understanding the structural aspects of Pth is like having a blueprint for a machine that gives us insights into how it functions and how we might be able to tweak its operation. The future of Pth research holds promise for developing new treatments for a wide range of diseases. Bacterial infections that rely on protein synthesis could be susceptible to drugs that interfere with Pth’s function. By combining expertise in structural biology, biochemistry, and clinical research, we can better understand how Pth works and how it might be targeted for therapeutic purposes. This interdisciplinary approach will be crucial for translating basic research findings into clinical applications that benefit patients.

In conclusion, studying Pth is not just about unraveling the mysteries of a single molecule; it is about unlocking new possibilities for designing broad-spectrum antimicrobial agents. As we continue to explore the intricacies of Pth and its role in cellular processes, we are paving the way for innovative therapeutic strategies that could transform medicine and improve human health.
